# Induction of DNA-mediated immune responses by bacterial extracellular vesicles results in control of murine norovirus infection

**DOI:** 10.1080/19490976.2026.2624171

**Published:** 2026-02-05

**Authors:** Guanqi Zhao, Chanel A. Mosby-Tourtellot, Javier Rosero, Alexander C. Schultz, Elsa Khan, Othmane Elamrani, Mariola J. Ferraro, Peter E. Kima, Melissa K. Jones

**Affiliations:** a Microbiology and Cell Science Department, IFAS, University of Florida, Gainesville, FL, USA

**Keywords:** Norovirus, murine norovirus, viral pathogenesis, bacterial extracellular vesicles, commensal bacteria, Enterobacter cloacae, Bacteroides thetaiotaomicron, microbiome, STING, TLR9, innate immunity, macrophage

## Abstract

Commensal bacteria have been a centerpiece for understanding interkingdom impacts on viral replication. Multiple groups have investigated the roles commensal bacteria played in regulating enteric virus infection and it has been found that the mechanisms through which this regulation occurs varies between the viruses and bacteria explored. For noroviruses, commensal bacteria enhance or suppress viral infection in a region-dependent manner. Recently, it was found that the extracellular vesicles (EVs) produced by commensal bacteria can suppress norovirus infection. In this study, we used murine norovirus (MNV) to probe the immunological mechanisms induced by bacterial EVs. Global analysis of gene expression pointed to induction of cytosolic DNA pathways; thus, we evaluate the DNA content packaged within the bacterial EVs and DNA-sensing pathways that activate type I interferons (IFN), including STING and TLR9. Our results showed that loss of *sting* or *tlr9*, significantly decreased IFNβ production and recovered MNV replication in the presence of bEVs. Collectively, these data demonstrated bEVs of certain gram-negative bacteria can initiate antiviral DNA-mediated type I IFN production pathways and that these pathways are involved in the suppression of MNV replication. These findings expose novel mechanisms through which the native microbiota aids the host in controlling an enteric viral infection and offers a fresh perspective on interkingdom host‒microbiota interactions.

## Introduction

Bacterial-derived extracellular vesicles (bEVs) have emerged as pivotal elements in microbiological research and offer a wealth of insight into host‒microbe interactions. All bacteria generate bEVs using a variety of biogenesis pathways that vary based on bacterium and growth conditions.^1-3^ The size of Gram-negative bEVs usually ranges from 25 to 250 nm, and these bEVs can carry multiple functional components from the parental bacteria, including lipopolysaccharide (LPS), phospholipids, DNA and/or RNA.^4^^,^^5^ Because of this, bEVs from both pathogenic and commensal bacteria are capable of immune modulation through a variety of pathways.^6-8^

Commensal bacteria modulate infection of all enteric viruses investigated to date, including noroviruses.^9^^,^^10^ Previous research has shown that noroviruses attach to commensal bacteria, and this attachment leads to changes in gene expression that have metabolic and functional impacts on the bacteria cells.^11^ One such change resulting from this interaction is that commensal bacteria increase bEV production. Interestingly, infection of mice with MNV also leads to increased bEV concentrations in stool, indicating a biological function for bEVs in the gut during viral infection.^11^ Further supporting this hypothesis, investigations into the impact of bEVs from Gram-negative and gram-positive bacteria on *in vitro* replication of MNV reveal that all suppress viral replication by priming antiviral responses.^12^^,^^13^ In addition to suppressing MNV replication, bEVs also inhibit influenza virus replication *in vitro* and *ex vivo*^14^ and control systemic replication of RNA and DNA viruses.^15^

Despite this common ability to suppress viral replication, the mechanisms behind bEV control of viral infection varies based on both the virus being targeted and the bEVs involved. For example, bEVs derived from pathogenic bacteria inhibited influenza replication via the TLR4-TIRF axis,^14^ while commensal bEVs injected intravenously in mice suppressed HSV-1 and VSV replication in circulation through systemic STING-induced type I interferon (IFN).^15^ Moreover, *in vitro*, the suppression of MNV infection by bEVs from the Gram-positive bacterium *Lactobacillus johnsonii* occurred through the induction of OAS pathways via TLR3.^13^ Based on the varied immune responses induced by different vesicle types, we set out to determine how bEVs from two Gram-negative constituents of the intestinal microbiome influenced MNV replication. Based on our previous findings that bEVs from the commensal bacteria *Enterobacter cloacae* and *Bacteroides thetaiotaomicron* contain DNA and induce type I IFN responses,^11^^,^^12^ we hypothesized that these vesicles would generate antiviral immune responses via DNA-mediated pathways like STING. Indeed, even from early time points, bEVs increased the production of key immune markers like *ifit1*, *irf1*, *isg15,* and *nfkb*. To gain a more complete view of the immunomodulated state induced by bEVs, we performed RNA sequencing and confirmed that DNA-sensing pathways (among others) are activated upon bEV exposure. We next confirmed the expression of key proteins along the STING pathway and used knockout cell lines to confirm the importance of *sting* in mediating type IFN production. We also discovered that another DNA-sensing pathway, *tlr9*, plays a role in suppressing MNV replication. Together, these data confirmed that DNA packaged by bEVs from the commensal bacteria *E. cloacae* and *B. thetaiotaomicron* is responsible, in part, for bEV-mediated suppression of MNV replication. Ultimately, this work provides a new perspective for studying human norovirus (HNoV) pathogenesis and a potential pathway for commensal bacteria to aid immunocompetent hosts in suppressing viral replication in the gut.

## Results

### Pretreatment of commensal bEVs suppress MNV replication *in vitro*

**Figure 1.**
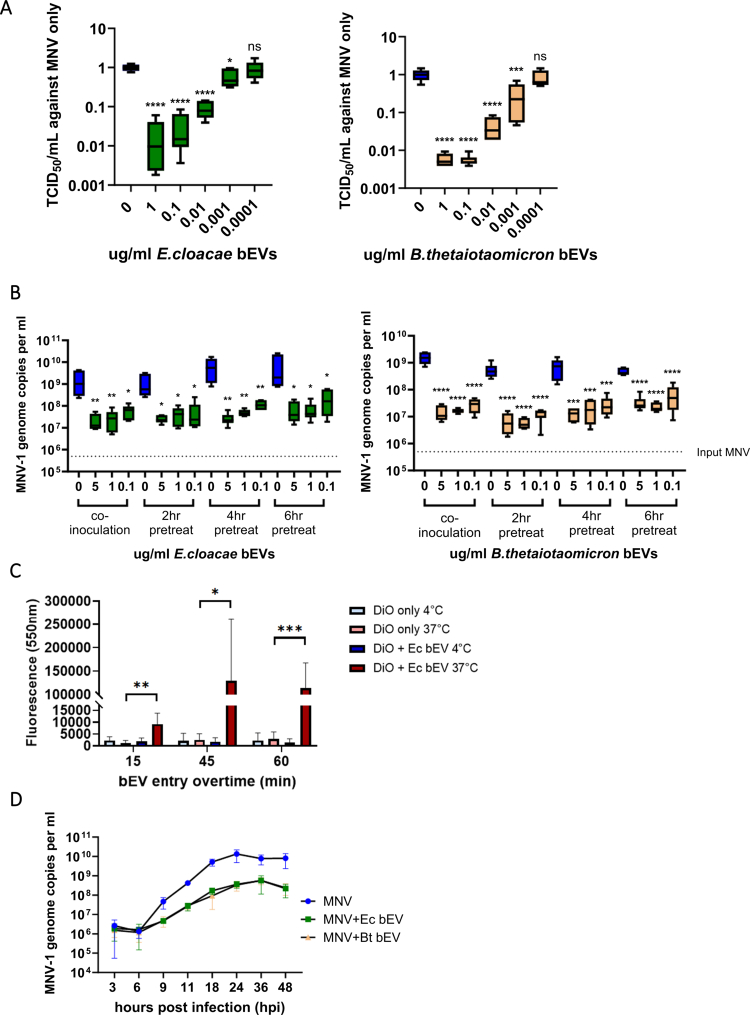
Early and sustained impact of commensal bEVs on cellular entry and MNV replication in RAW 264.7 macrophages. (A) *E. cloacae* and *B. thetaiotaomicron* bEVs (1, 0.1, 0.01, 0.001, and 0.0001 µg/ml) were inoculated with MNV on RAW macrophages. After 18 h, the infection supernatant was collected for TCID_50_ analysis. *n* = 6. (B) RAW macrophages were pretreated with *E. cloacae* or *B. thetaiotaomicron* bEVs (5, 1, or 0.1 µg/ml) for 2, 4, and 6 h prior to MNV infection. At 18 hpi, supernatants were collected for MNV quantificaiton by RT–qPCR and Compared to bEV-MNV co-inoculation. *n* = 6. (C) RAW 264.7 macrophages cells were inoculated with either DiO only, or DiO +5 µg/ml bEV for 15, 45, and 60 min under either 4 or 37 °C. bEV entry profile were recorded by calculating the GFP pixel intensity within a 10 µm radial extension from nucleus. (D) RAW cells were coinfected with bEVs + MNV and supernatants collected at 3, 6, 9, 11,18, 24, 36, and 48 hpi. MNV titer was determined by RT–qPCR. *n* = 6. *p < 0.05; **p < 0.01; ***p < 0.001; ****p < 0.0001.

We previously found that *in vitro* coinoculation of macrophages with MNV and bEVs from both pathogenic and commensal Gram-negative bacteria resulted in suppression of viral titers at the peak of viral replication.^12^ To further explore the suppressive nature of Gram-negative bEVs, we evaluated the dosing effect of vesicles from *Enterobacter cloacae* and *Bacteroides thetaiotaomicron*. These bacteria were chosen because they represent major (*B. thetaiotaomicron*) and minor (*E. cloacae*) constituents of the intestinal microbiome, which colonize different regions of the intestinal tract (colon and small intestine, respectively).^16^^,^^17^ Furthermore, previous work has shown that *E. cloacae* facilitates human norovirus infection of B cells *in vitro.*^9^^,^^18^ RAW 264.7 macrophages were treated with serial dilutions of these bEVs and MNV replication was measured at 18 hpi. bEVs from both bacteria were able to reduce MNV replication in a dose-dependent manner (Figure 1A). Considering that the impact of bEVs on viral replication has been previously coupled with changes in immunological responses,^15^^,^^19-22^ we tested the hypothesis that pretreatment of cells with *E. cloacae* and *B. thetaiotaomicron* bEVs prior to MNV infection would induce antiviral immune responses resulting in further suppression of MNV replication. Interestingly, all of the pretreatment conditions failed to improve MNV suppression compared to bEV coinoculation (Figure 1B). One possible explanation for this observation is that the bEVs enter cells swiftly and initiate antiviral responses immediately after internalization. Indeed, previous research has shown that Gram-positive bEVs enter *β* islet cells in as little as 15 min and bEVs from *B. thetaiotaomicron* can enter epithelial cells within 2 h.^23^^,^^24^ We had previously shown that *E. cloacae* bEVs entered RAW 264.7 macrophages after at least 60 min of incubation, but early time points were not examined. Therefore, DiO-labeled *E. cloacae* bEV entry into RAW 264.7 macrophages was quantified at earlier timepoints. Results showed that vesicle uptake could be seen as early as 15 min after inoculation (Figure 1C, Fig. S1), supporting the hypothesis that bEVs have a very rapid impact on cellular immune responses that far precedes viral replication. In addition, little to no fluorescence was detected in cells incubated on ice for any of the time points tested, indicating that bEV entry into macrophages occurs in an energy dependent manner (Figure 1C).

To evaluate the early and extended impact of bEVs on MNV infection, we next quantified viral replication over time. These assays revealed that both *E. cloacae* and *B. thetaiotaomicron* bEVs began suppressing viral replication as early as 9 hpi (Figure 1D). Interestingly, while viral titers increased in bEV-treated samples, they remained significantly lower than untreated infected cultures for the duration of the experiment. Since viruses remain in the culture and bEVs do not have a negative impact on cell viability (Fig. S2A), it is possible the effect of bEVs on host immune responses could wane and allow for a resurgence of viral replication. However, our results showed the bEVs continued to suppress MNV replication through the 48 h time point (Figure 1D). Together, these data indicate that bEVs have an early and lasting impact on immunological signaling, resulting in sustained reductions in MNV replication.

### Changes in gene expression after *E. cloacae* bEV inoculation in RAW 264.7 macrophages

**Figure 2.**
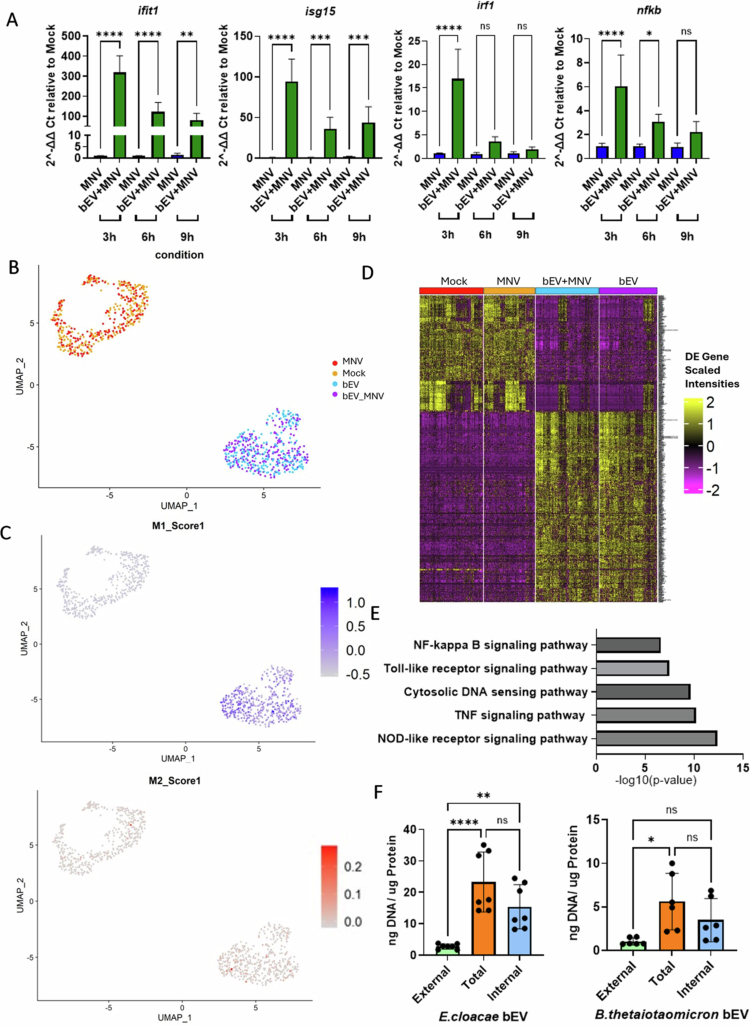
Impact of bEVs on gene expression and predicted pathway induced by bEVs. (A) RAW cells were coinfected with bEVs (1 µg/mL) and MNV (MOI = 5), and gene expression were measured at 3, 6, and 9 hpi using RT–qPCR. (B) UAMP clustering using the 1 K cDNA library. (C) M1 and M2 polarization across UMAP clustering. (D) Heatmap showing all DEG expression of all four conditions (Mock, MNV, EcbEV and EcbEV + MNV) against model murine genome GRCm38. *P* adjusted value < 0.05, log_2_ FC = 0.5. (E) DAVID functional analysis using KEGG pathway annotation. The graph shows a list of selected pathways with low modified Fisher's exact *p* values. (F) dsDNA content on bEVs was measured using Quant-iT PicoGreen assay. *n* = 6. *p < 0.05; **p < 0.01; ***p < 0.001; ****p < 0.0001.

To gain an understanding of which immune responses are activated by bEVs to counteract MNV replication we used time course experiment samples to measure expression of interferon-stimulated genes (ISGs). We first quantified *ifit1*, *isg15*, *irf1,* and *nfkb* expression after exposure to *E. cloacae* bEVs as these genes contribute to suppression of norovirus replication.^25^^,^^26^ RT–qPCR data revealed that, while the MNV-only group failed to stimulate significant ISG gene production at early time points, *E. cloacae* bEV-inoculated samples displayed elevated ISG gene expression as early as 3 hpi (Figure 2A). We next performed single-cell RNA sequencing to get a more complete view of differential gene expression and to determine if individual cells that had taken up *E. cloacae* bEVs could be identified. Two cDNA libraries were sequenced: one with 1 K cells and the other with 5 K cells. Both libraries were assembled and the 1 K library was used for downstream analysis due to improved sensitivity resulting from a greater sequencing depth. At 6 hpi, UMAP analysis identified five different subgroups (Fig. S3A) and showed that mock and MNV-treated cells clustered away from the bEV-treated groups (Figure 2B). Given the suppression of MNV replication, it is likely that inflammatory signals are induced which could lead to changes in macrophage subtype. Using the current UMAP analysis (Figure 2B), M1 and M2 populations were labeled. M1 (proinflammatory) and M2 (anti-inflammatory) denote distinct macrophage activation states. Comparing the clusters with and without bEVs, the bEV-exposed cells were dominated by M1 populations while the amount of M2 cell types was similar between the bEV-exposed and bEV-unexposed groups (Figure 2C). This finding is further supported by statistical analysis of the data demonstrating that significant differences in M1 populations are present between the bEV-exposed and unexposed populations (Fig. S3B). Unsurprisingly, there is also a difference in the number of M1 macrophages in MNV-exposed cells compared to mock exposed cells, although the M1 populations remain significantly larger in bEV-exposed cells (Fig. S3B). Ultimately, our scRNA-seq data indicate that stimulation with bEVs causes RAW macrophages to exhibit a transcriptional shift toward the proinflammatory M1 phenotype. Since bEVs can package both DNA and RNA and we have previously demonstrated that *E. cloacae* genomic DNA is associated with its bEVs,^11^ we attempted to identify cells that had taken up bEVs using scRNA-seq. The 1 K cDNA libraries were screened against the *E. cloacae* genome for the presence of bacterial RNA, but bacterial sequences were not detected in any samples, possibly due to the low abundance bacterial RNA carried by bEVs. In light of this, the data was analyzed as bulk RNA-sequencing data and we used a model murine genome GRCm38 (mm10) to identify all differentially expressed genes (DEGs) in the samples. As expected, multiple ISGs (e.g. *rsad2*, *ifit1*, *mx1*, *cxcl10*, *mir155hg*, etc.) were identified from the top 50 most variable gene list (Supplemental Table 1).

Similar to UMAP analysis, mock and MNV-treated groups had comparable expression patterns and the bEV-only and bEV + MNV groups also had a presented high resemblance (Figure 2D). We compared DEGs between mock and bEV-only groups and found 454 upregulated DEGs in the bEV-only population (adjusted *p* < 0.05 and log_2_ fold change of 0.5). DAVID (Database for Annotation, Visualization, and Integrated Discovery) functional analysis was used prevalently in efficiently identifying biologically relevant pathways from differentially expressed genes.^27^^,^^28^ Therefore, DAVID was used to predict the topmost mediated pathway annotated by KEGG database. Cytosolic DNA sensing pathway was among the top five pathways identified (Figure 2E), indicating that *E. cloacae* bEV-associated DNA likely plays a role in early immune activation in macrophages and may be involved in bEV-mediated suppression of MNV replication. Additionally, by performing enrichment analysis on downregulated DEGs, both “bEV vs mock” and “bEV + MNV vs MNV only” indicated that bEV treatment had a negative impact on pathways related to decreased cell proliferation ^29^ (Fig. S4). This aligns with our MTS assay data (Fig. S2A) and previous published experiments which show that bEVs improve cell viability and reduce cytotoxicity upon MNV infection.^12^

bEV cargo composition and content has been analyzed for many bacterial species,^30^^,^^31^ and DNA content has been shown to be packaged within vesicles for many gram-negative bacteria.^32^ Since cytosolic DNA-sensing pathways were differentially expressed in bEV-treated cells, we quantified the amount of DNA associated with *E. cloacae* and *B. thetaiotaomicron* bEVs. The total, internal, and external DNA content of the bEVs was measured and revealed that dsDNA is present both externally and internally for *E. cloacae* and *B. thetaiotaomicron* bEVs, with greater concentrations of dsDNA detected inside the vesicles (Figure 2F). These data further support the RNA-seq data, indicating that cytosolic DNA-sensing pathways are activated by bEVs. Since the STING pathway plays vital roles in sensing DNA and priming of antiviral response, we first measured *sting-1* gene expression on RAW cells after bEV + MNV treatment and found that treatment induced *sting-1* expression at early infection time points (Fig. S5A). While packaged dsDNA likely induces STING signaling,^15^ the presence of externally bound DNA indicates that other DNA-mediated immune responses may also be stimulated by these bEVs. Specifically, if bEVs enter via endocytosis, externally bound DNA may stimulate endosomal TLR9. Analysis of *tlr9* also showed upregulated gene expression at early time points following exposure to bEV + MNV (Fig. S5A), indicating that this DNA-sensing pathway might also be involved in MNV suppression.

### Changes in protein expression after *E. cloacae* bEV inoculation in RAW 264.7 macrophages

**Figure 3.**
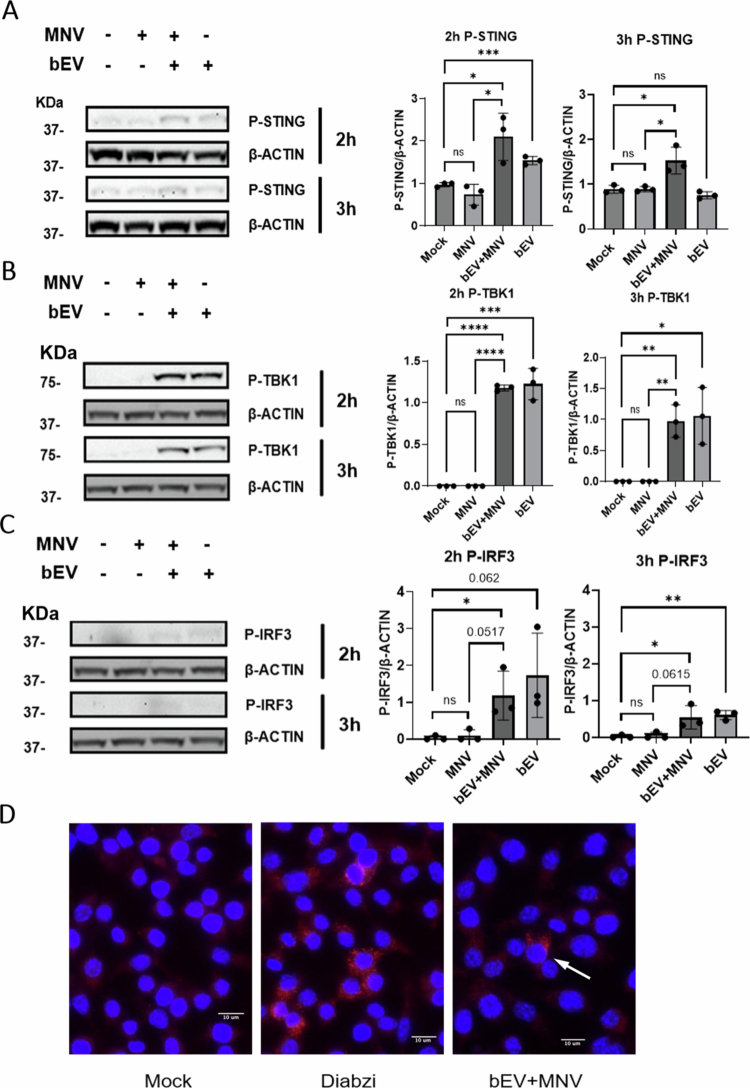
Protein expression of key STING pathway proteins. Induction of key phophorylated proteins along the STING pathway (A) STING, (B) TBK1, and (C) IRF3 was observed at 2 and 3 hpi. *β*-actin was used as a loading control. Quantification of band intensity was achieved using FIJI software as protein of interest/*β*-actin. *n* = 3. *p < 0.05; **p < 0.01; ***p < 0.001; ****p < 0.0001. (D) RAW 264.7 cells seeded on cover slips treated with Diabzi (positive control), mock virus, *B. thetaiotaomicron* bEV + MNV, or MNV alone (image not shown) then fixed. DAPI (blue) was used to label the cell nucleus and *P*-STING (Alexa 594) activity was visualized by red puncta adjacent to the nucleus. Images were taken under 40× on Keyence BZ-X810 fluorescentfluorescence microscope.

We next evaluated expression of key proteins along DNA-mediated immune pathways. The STING signaling pathway can recognize both host-derived or microbial dsDNA, thus activating the downstream antiviral type I IFN production.^33^ Although type III IFN also serve as a downstream marker for STING activation^34^^,^^35^ and have previously been reported to regulate MNV infection of epithelial cells,^10^^,^^36^ *ifnl2*, *ifnl3* were not expressed in our RAW 264.7 macrophages cells and were also not induced by bEVs and thus were not included in our downstream analysis (Fig S5B). Western blot analysis showed that bEV exposure increased phosphorylated STING (*P*-STING) expression starting at 2 hpi (Figure 3A). After phosphorylation, STING translocates from the ER to the Golgi body and recruits TBK1 for phosphorylation. We also observed increased *P*-TBK1 and 2 and 3 hpi (Figure 3B). The next step in the pathway is *P*-TBK1 catalyzation of IRF3 phosphorylation,^33^ and this was also observed after bEV treatment (Figure 3C). The presence of all three phosphorylated proteins (STING, TBK1, and IRF3) confirms bEV activation of the STING pathway. Interestingly, only the bEV + MNV condition induced noticeable STING phosphorylation (*P*-STING) at the time points examined whereas samples treated with only bEVs showed insignificant increases in protein expression (Figure 3A). Visible *P*-STING expression for bEV-treated sample was weaker than expected, so an immunofluorescence assay (IFA) was performed to confirm the presence of *P*-STING after bEV treatment. The appearance of red punctate, similar to the positive control (Diabzi) confirmed that the active components of the STING pathway were expressed upon bEV exposure (Figure 3D).

### DNA-mediated pathways aid in mediating antiviral responses upon *E. cloacae* bEV exposure

**Figure 4.**
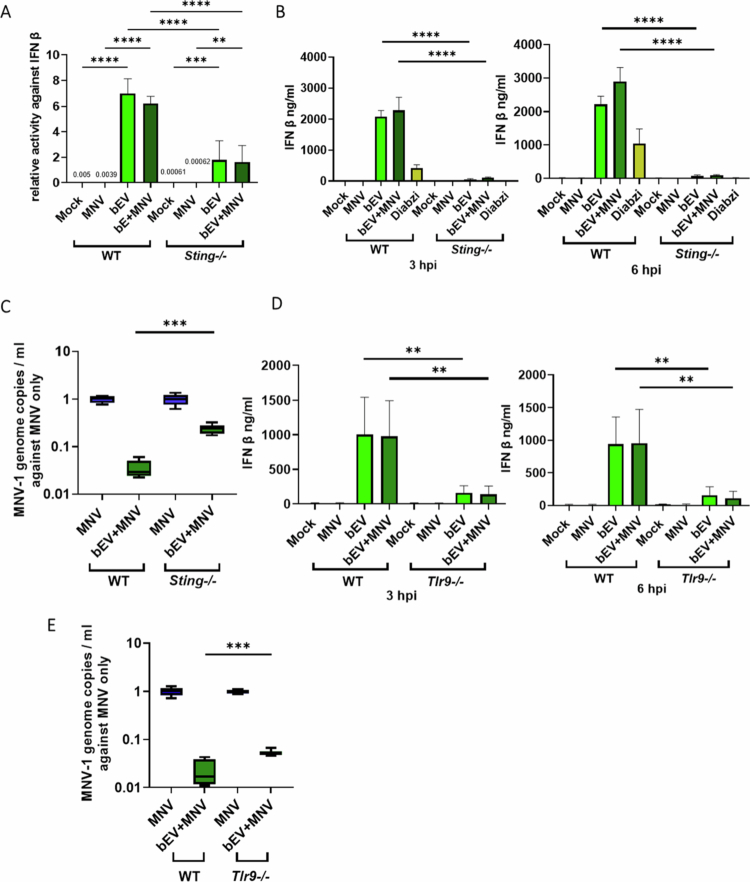
Impact of *sting* and *tlr9* on RAW macrophages' antiviral ability. (A) Luciferase activity was detected at 6 hpi. y axis was denoted as ratio of unit of activity against IFNβ treated only as control. Diabzi was used as a positive control for STING induction. *n* = 6. (B,D) IFNβ expression was measured by ELISA. RAW cells were treated with bEVs (1 µg/ml), and the supernatants were collected at 3 and 6 hpi. Diabzi was used as a STING agonist. *n* = 6. (C, E) At 18 hpi, the infection supernatant was collected for RNA extraction followed by RT–qPCR. y axis was denoted as ratio of MNV genome copies per mL against MNV only sample. *n* = 6. **p < 0.01; ***p < 0.001; ****p < 0.0001.

To validate bEV induction of STING and the role of this pathway in suppression of MNV replication, we employed a *sting*^-/-^ RAW cell line with an ISG54 luciferase tag. As discussed above, bEV treatment improves cell viability in both infected and uninfected WT cells (Fig. S2A). Their impact on *sting*^*-/-*^ cells was also examined, and although bEV treatment offered some improvement in cell viability, the degree of viability increase was not as dramatic as was observed with WT cells (Fig. S2B). When evaluating the impact of bEVs on immune signaling, RAW *sting*^*-/-*^ cells produced significantly less ISG54 compared to WT cells after bEV exposure (Figure 4A). However, ISG54 was still detectable in the *sting*^*-/-*^ cells indicating that activation of antiviral pathways other than STING are involved in bEV induction of ISG54. Since viability is maintained in these cells after bEV treatment (Fig. S2B), the production of type I IFN, which plays an essential role in inhibiting the replication of MNV replication, was examined by measuring IFNβ production.^26^^,^^37^ At 3 and 6 hpi, bEV treatment led to substantial IFNβ production which was absent in both mock and MNV conditions at these early time points (Figure 4B). In contrast, bEV treatment of RAW *sting*^*-/-*^ cells resulted in significantly lower IFNβ production, indicating that loss of the STING pathways hinders the ability of bEVs to suppress this key IFN activation protein (Figure 4B). We next quantified MNV replication in *sting*^*-/-*^ cells to confirm that this pathway was involved in the observed bEV suppression of replication. At 18 hpi, loss of the STING pathway led to partial rescue of MNV infection (Figure 4C), indicating that, while STING is involved in bEV suppression of MNV, other immune pathways are also induced by the vesicles to suppress infection as was indicated by ISG54 suppression data (Figure 4A).

In light of the likely contribution of other immune pathways and since STING is not the only pathway stimulated by DNA to produce type I IFN, we turned to another DNA-primed antiviral pathway. *Moraxella catarrhalis* derived EVs can initiate B cell activation via the TLR9 endosomal receptor.^38^ We found that, similar to *sting* expression, *tlr9* expression is also upregulated early after bEV treatment (Fig. S5A). Therefore, to determine if our bEVs can activate TLR9 to suppress MNV infection, we performed the bEV-MNV coinfection assay using iBMDM WT and iBMDM *tlr9*^*-/-*^ cells. Similar to what was observed with *sting*^*-/-*^ macrophages, bEV-treatment of *tlr9*^*-/-*^ cells showed a significant reduction in IFNβ production (Figure 4D) and a partial rescue of MNV suppression (Figure 4E), indicating that both the STING and TLR9 pathways are involved in bEV suppression of MNV. These data also reveal that the STING pathway is likely a primary mediator of the antiviral effects of bEVs with TLR9 playing a lesser role and indicate that other, currently unidentified pathway(s), are also involved in suppression of MNV replication.

## Discussion

Commensal bacteria have a complex relationship with enteric viral infection. In some instances these bacteria promote viral infection by enhancing virion stability or inducing immune tolerance via LPS.^39-41^ In other circumstances, they suppress viral replication via bile acid-primed type III IFN, but in a region-specific and sometimes cell-specific manner.^10^^,^^36^ More recently, investigations into the ability of bacterial EVs to shape the host immunological state during viral infection have been explored. bEVs from pathogenic bacteria can block influenza virus infection *in vitro* and *ex vivo* via the TLR4-TIRF axis,^14^ and bEVs from commensal bacteria can induce systemic immune responses to suppress viral replication in the bloodstream through cGAS-STING.^15^ To date, the immune mechanisms involved in bEV modulation of enteric viral infection have not been reported. To address this knowledge gap, we investigated the effects of commensal bEVs on RAW macrophages during MNV infection since these are the primary and most prolific cell type infected by the virus *in vivo* and *in vitro*.

Our studies revealed that bEVs from both *E. cloacae* and *B. thetaiotaomicron* species reduced MNV replication in a dose-dependent manner when coinoculated with macrophages. Interestingly, pretreatment of macrophages with bEVs did not further enhance suppression of viral replication compared to co-inoculation, suggesting that bEVs rapidly initiate antiviral immune responses after cellular uptake. Supporting this, labeled bEVs were shown to enter macrophages within 15 min, and early antiviral effects were observed as soon as 3–9  hpi, with strong and prolonged suppression of MNV replication lasting up to 48 h despite continued viral presence. While the current studies focused on macrophage infection since it is a primary cellular target of acute MNV infection, future experiments will explore the role of bEVs in modulating acute and persistent MNV infection using other norovirus strains and cellular targets such as dendritic cells, B cells, T cells, and epithelial cells.

Focusing on ISGs and cytosolic DNA-sensing pathways, we next evaluated the immune responses activated by *E. cloacae* bEVs. RT–qPCR revealed that bEV-treated cells exhibited early induction of key ISGs, whereas MNV-infected cells alone failed to elicit a strong immune response at these same early time points. The lack of MNV induction of ISGs was expected as viral replication is not typically detected until 6‒9 hpi, and the first hints of ISG at 4 hpi have been previously reported.^42^^,^^43^ Thus, to capture a complete view of the immunomodulation carried out by bEVs prior to immune stimulation by MNV we performed scRNA-seq using a 6 hpi timepoint. As expected, distinct clustering of mock and MNV-only samples occurred with another cluster containing bEV-only and bEV + MNV-treated samples, further confirming that bEV treatment significantly altered cellular gene expression apart from MNV replication at early time points. Functional pathway analysis identified the cytosolic DNA-sensing pathway as a key mediator of *E. cloacae* bEV-induced immune activation, supporting the hypothesis that bEV-associated DNA contributes to antiviral defense. The presence of external and internal DNA for both *E. cloacae* and *B. thetaiotaomicron* bEVs further supported this hypothesis and also indicated that TLR9 or other DNA-mediated immune pathways are also activated by these bEVs. The induction of *P*-STING, *P*-TBK1, and *P*-IRF3 was observed and confirmed activation of the STING pathway by our commensal bEVs. Notably, *P*-STING expression was more pronounced in the bEV + MNV condition compared to bEV treatment alone, suggesting a synergistic effect between these bEVs and viral infection in enhancing immune activation of this pathway even in the absence of viral induction of gene expression. Immunofluorescence further validated these findings, showing clear *P*-STING localization upon bEV exposure, despite the relatively weak signals observed in Western blots. Overall, these results demonstrated that *E. cloacae* and *B. thetaiotaomicron* bEVs rapidly activate innate immune responses through STING phosphorylation. The detection of both internal and external DNA in bEVs indicates that these vesicles have the ability to stimulate multiple DNA-sensing pathways. Given that these vesicles enter through an energy-dependent manner, such as endocytosis,^23^^,^^24^ this provides the opportunity for the vesicles to stimulate endosomal TLR9 receptors. Moreover, once released from the endosome, the release of vesicle contents into the cytoplasm allows stimulation of the STING pathway. Therefore, the role of each of these pathways in bEV suppression of MNV infection was investigated.

To further investigate the role of the STING and TLR9 pathways in bEV-mediated suppression of MNV replication, knockout cell lines and siRNA interference were utilized. STING-deficient (*sting-/-*) RAW cells exhibited reduced but still detectable ISG54 expression and activation of alternative antiviral pathways. IFNβ production was significantly lower in *sting-/-* and *tlr9-/-* macrophages, and both knockout cell lines showed partial rescue of MNV replication, indicating that both pathways contribute to bEV-induced antiviral immunity. These findings collectively suggest that *E. cloacae* and *B. thetaiotaomicron* bEVs engage multiple immune pathways, with additional yet unidentified mechanisms contributing to their antiviral effects and highlight the complexity of bEV-driven immune activation.

In conclusion, this work highlights the potential of bEVs from commensal bacteria to rapidly and persistently suppress MNV replication by triggering early antiviral immune responses. Our previously published work has shown that bEVs from *Salmonella enterica* and *Lactobacillus johnsonii* also suppress MNV replication, indicating a widespread role for pathogenic and commensal bEVs in controlling acute infection.^12^^,^^13^ Interestingly, the *L. johnsonii* bEVs did not suppress MNV replication through STING but rather through the TLR3-OAS axis, indicating that commensal bEVs employ a multipronged immunological approach to signal to the host regarding viral infection.^13^ In addition, similar to *L. johnsonii* bEVs, our data suggest that *E. cloacae* bEV uptake occurs swiftly and leads to an immediate immunological impact, explaining the lack of enhanced suppression with pretreatment. Overall, commensal bEVs represent a promising avenue for modulating host antiviral defenses and controlling viral infections. Indeed, bEVs are already being tested as potential vaccine candidates for both bacterial and viral infections. Therefore, understanding the specific components contained in these vesicles and the immune pathways they stimulate are paramount to the development and implementation of this new technology.

## Methods

### Cell culture

All cell lines (RAW 264.7 (ATCC), RAW-Lucia™ ISG WT and *Sting-/-* (InvivoGen), iBMDM WT and *tlr9-/-* (BEI resources)) were incubated in complete DMEM with 10% FBS, 100 U/mL pen/strep.^12^ Cells were cultured at 37 °C at 5% CO_2_.

### Bacterial culture and bEV isolation

*E. cloacae* was cultivated in Luria Bertani (LB) medium with 1% NaCl under aerobic conditions at 37 °C with continuous shaking (220 rpm). *B. thetaiotaomicron* was cultured in conditioned brain and heart infusion broth, supplemented with 0.001% hemin at 37 °C under anaerobic conditions using anaerobic chambers and gas generator packs. At the late log phase, the cultures were harvested, and bEVs were isolated as previously described.^11^ bEVs were inoculated onto LB agar to confirm the absence of bacterial contamination and stored at 4 °C for no longer than 2 weeks.

### Generation of MNV

The plasmid pSPMNV-1. CW3 was a kind gift from Dr. Stephanie Karst (University of Florida) and was used for generating recombinant murine norovirus-1 (MNV-1) as previously described.^44^ HEK293T cells were transfected with the plasmid and incubated overnight. The supernatant was then used to infect RAW 264.7 cells at an MOI of 5. A mock virus was generated in parallel using 293 T cells transfected without plasmid. This mock was used as a control for all infection assays.

### *In vitro* treatment assays

RAW 264.7 cells were seeded at 1 × 10^6^ cells per mL in 12-well plates. After overnight incubation, the cells were inoculated with mock inoculum, MNV (MOI = 5), MNV plus bEVs, or bEVs only. The cells were incubated for 1 h at 37 °C, after which the inoculum was removed, and the cells were washed twice with dPBS. The supernatants were collected at the noted time points and used for RT–qPCR analysis.

### RT–qPCR MNV detection

Following manufacturers' instructions, the supernatant from infection was treated using the Quick RNA MiniPrep kit (Zymo Research). RNA was converted to cDNA (M-MLV RT) and quantified using qPCR. cDNA combined with MNV forward and reverse primer.^9^ PowerUp SYBR Green Master Mix and genome copies quantified using a QuantiStudio3 system. Results were converted to MNV genome copies per sample using a standard curve generated with each plate, which was based on linearized pSPMNV-1.CW3 plasmid.^12^

### TCID_50_ assay

TCID_50_ assay was performed as previously described.^45^^,^^46^ 3 × 10^4^ per well RAW macrophages were seeded in a 96 well plate overnight. The next day the media was removed, and cells were inoculated with serial dilutions of sample and incubated for 7 d. On day 8, each well was examined for CPE and TCID_50_/ml was determined by Reed-Muench method.

### bEV labeling and cellular entry

bEVs were fluorescently labeled with Vybrant™ DiO for 30 min at 37 °C. Free floating dye was removed by using Amicon® Ultra-4 centrifugal filter, and the samples were washed with PBS for three times. Labeled bEVs were then added to RAW 264.7 cells and incubated either on ice or at 37 °C for 15, 45, and 60  min. The cells were then fixed with 4% paraformaldehyde (PFA) and nucleus were stained using DAPI. Fluorescence microscopy images were taken using Biotek Cytation 5 imager.

### RT–qPCR for gene expression

RNA was isolated from cell supernatants following the manufacturers' instructions (Zymo Research). To remove the genomic DNA, RNA samples were treated with Turbo Dnase free kit. Briefly, samples were mixed with 10× Dnase buffer and 3 U of DNase I and DNase were inactivated after 30 min at 37 °C water bath. qPCR was performed for the noted genes and *gapdh* was also measured for each plate as reference gene. All result were normalized using the 2^^^^−ΔΔCT^ method.

### MTS viability assay

MTS assay was performed following the manufacturer's instructions (Abcam) as described previously.^12^ RAW 264.7 cells were seeded in a 96-well plate and inoculated with controls and samples of interest the next day. MTS working reagent was added for 2 h followed by OD measurement at 490 nm.

### Single-cell RNA sequencing

A single-cell suspension was prepared following the manufacturer's protocol (Parse biosciences). Two cDNA libraries with 1 K and 5 K cells were set up for downstream sequencing (Illumina NovaSeq). Fastq file processing was performed following the Parse pipeline. *Seurat* was used for quality control. Mm10 (Genome assembly GRCm38, NCBI) was selected as reference genome. Cells following these requirements are kept: >200 and <9500 genes, <9% mitochondrial contents, and gene count <150,000. UMAP was used to visualize data clustering. FindAllMarker function was used to identify unique markers which will be used for comparing one cluster to another. Published M1-associated (“*Il1a*”, “*Il1b*”, “*Tnf*”, “*Il6*”, “*Nos2*”, “*Cd86*”, “*Ccr7*”) and M2-associated (“*Mrc1*”, “*Fn1*”, “*Arg1*”, “*Retnla*”) genes set were curated from Jablonski et al. ^47^ For each sample, an enrichment score for the M1 and M2 gene sets was calculated using the AddModuleScore function in the Seurat R package. Polarization scores were plotted for each condition using violin plots generated by the *ggpubr* R package. The log fold change threshold was set at 0.5 and *p* adjusted value at <0.05. Upregulated genes were analyzed using DAVID functional enrichment analysis using KEGG pathway parameters.^48^^,^^49^

### PicoGreen assay

DNA content was quantified using the Quant-iT™ PicoGreen™ dsDNA Assay. External DNA content was measured by directly processing bEV samples. Total DNA content was measured by treating bEVs with GES lysis buffer. Internal DNA content was measured by treating bEVs with 3 U of TURBO™ DNase to remove externally bound DNA prior to lysis with GES buffer. Samples were prepped following the manual’s instructions and fluorescence read using Cytation 5 (Biotek) plate reader.

### Western blot analysis

Cells were lysed using NP40 buffer at desired time points. After centrifugation at 14,000 × g for 30 min the supernatant was collected and mixed with 4× XT sample buffer and 2-Mercaptoethanol and boiled at 95 °C. The samples were loaded on a XT criterion 4%–12% Bis-Tris acrylamide gel and then transferred to a nitrocellulose membrane. The membrane was washed with TBST and blocked with 5% nonfat milk. The proteins of interest were detected using primary antibodies at 1:1000 (Cell signaling technology: *P*-STING #D8F4W, *P*-TBK1 #D52C2, *P*-IRF3 #D601M, *β*-actin #13E5) and secondary Anti-rabbit IgG, HRP-linked Antibody at 1:3000 (Cell signaling technology). The blots were visualized using Clarity Western ECL substrate on the iBright imaging system. Band intensity was quantified using Image J studio.

### Immunofluorescence assay

Cover slips were placed in 6-well plates prior to seeding. Infection protocol proceeded as described above. At 2 hpi, cells were fixed with 2% paraformaldehyde (PFA) for 20 min at room temperature. Then, the PFA was quenched, and the cells were washed with PBS for 10 min. The cells were blocked for 15 min with 0.5% Triton X-100 plus 2% BSA, then washed 2× with PBS, and incubated with *P*-STING primary antibody (Cell Signaling Technology). Cells were washed with PBS, incubated with Alexa Fluor™ Plus 594 secondary goat anti-rabbit antibody (Invitrogen) and washed with PBS prior to mounting on slides and treating with DAPI. Images were taken using the BZ-X810 fluorescent microscope under 40×.

### ELISA assay

ELISA assay was performed by following manufacturer's instructions (Quantikine ELISA). Infection supernatant was collected from RAW 264.7 or iBMDMs infections and added onto a microplate coated with IFN-*β*. The enzyme conjugated monoclonal antibody was added followed by the substrate solution. Detection of color intensity was measured using Cytation 3.

### Luciferase assay

RAW-Lucia™ ISG cells WT (purchased from InvivoGen) and *sting-/-* (gift from Dr. Lei Jin, University of Florida) were infected with either MNV or MNV plus bEV. At 6 hpi supernatant was collected and mixed with QUANTI-Luc™ 4 Lucia/Gaussia reagent for flash luciferase detection.

### Statistical analysis

Data was analyzed using the GraphPad prism software. Error bars in all figures represent the standard errors of the mean, and *p*-values were calculated using unpaired two-tailed Student's t-tests (*p < 0.05, **p < 0.01, ***p < 0.001, ****p < 0.0001).

## Supplementary Material

Supplemental only_resub_final.docx Supplemental only_resub_final.docx

## Data Availability

Data will be made available in the publicly available repository Open Science Framework at osf.io using DOI 10.17605/OSF.IO/9BH4G.
