# Supplementary material for: Induction of DNA-mediated immune responses by bacterial extracellular vesicles results in control of murine norovirus infection
Source: Gut Microbes. 2026 Feb 5;18(1):2624171. doi: 10.1080/19490976.2026.2624171 (PMC12885434; doi:10.1080/19490976.2026.2624171)
Supplement: Supplemental only_resub_final.docx [file KGMI_A_2624171_SM4968.docx]

**Figure S1.** **DiO Fluorescence pixel intensity after 15, 45, 60 minutes of bEV inoculation.** Vybrant™ DiO labeled bEVs were inoculated on RAW cells for 15, 45 and 60 minutes prior to fixation and imaging using a Cytation5 imager. DAPI was used to label nucleus.


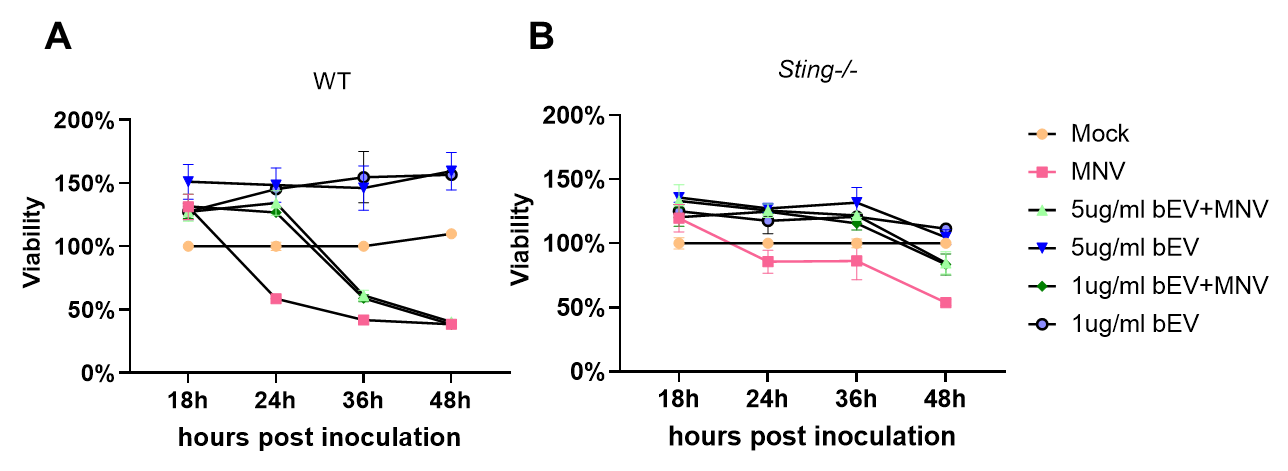


**Figure S2.** **Cell viability for WT and *Sting-/-* RAW macrophages from 18h to 48h post inoculation of MNV, bEV or bEV+MNV.** MTS assay was performed on RAW cells inoculated with MNV, *E. cloacae* bEV, and *E. cloacae* bEV+MNV. Cells were treated with MTS regent at 18h, 24h, 36h and 48h post infection. (A) WT RAW 264.7 macrophages. (B) *Sting-/-* RAW 264.7 macrophages. n=2.

.


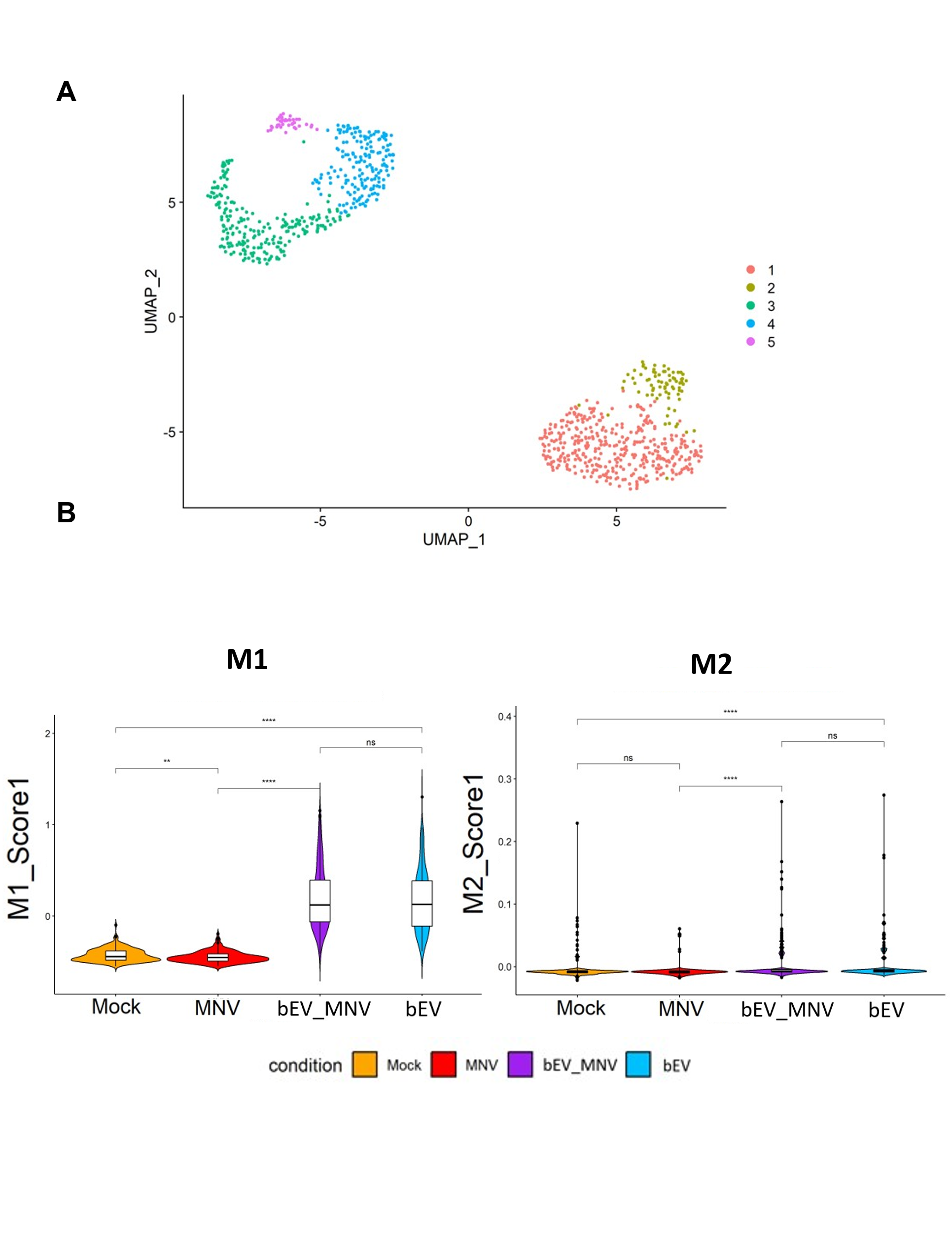


**Figure S3.** **UMAP analysis of macrophages treated with bEVs.** (A) Unsupervised UMAP clustering using 1k cDNA library from scRNA sequencing data. (B) Violin plot demonstrating M1 and M2 polarization across all four conditions (Mock, MNV, EcbEV and EcbEV + MNV) via the wilcox test. ** p < 0.01; **** p< 0.0001.

 
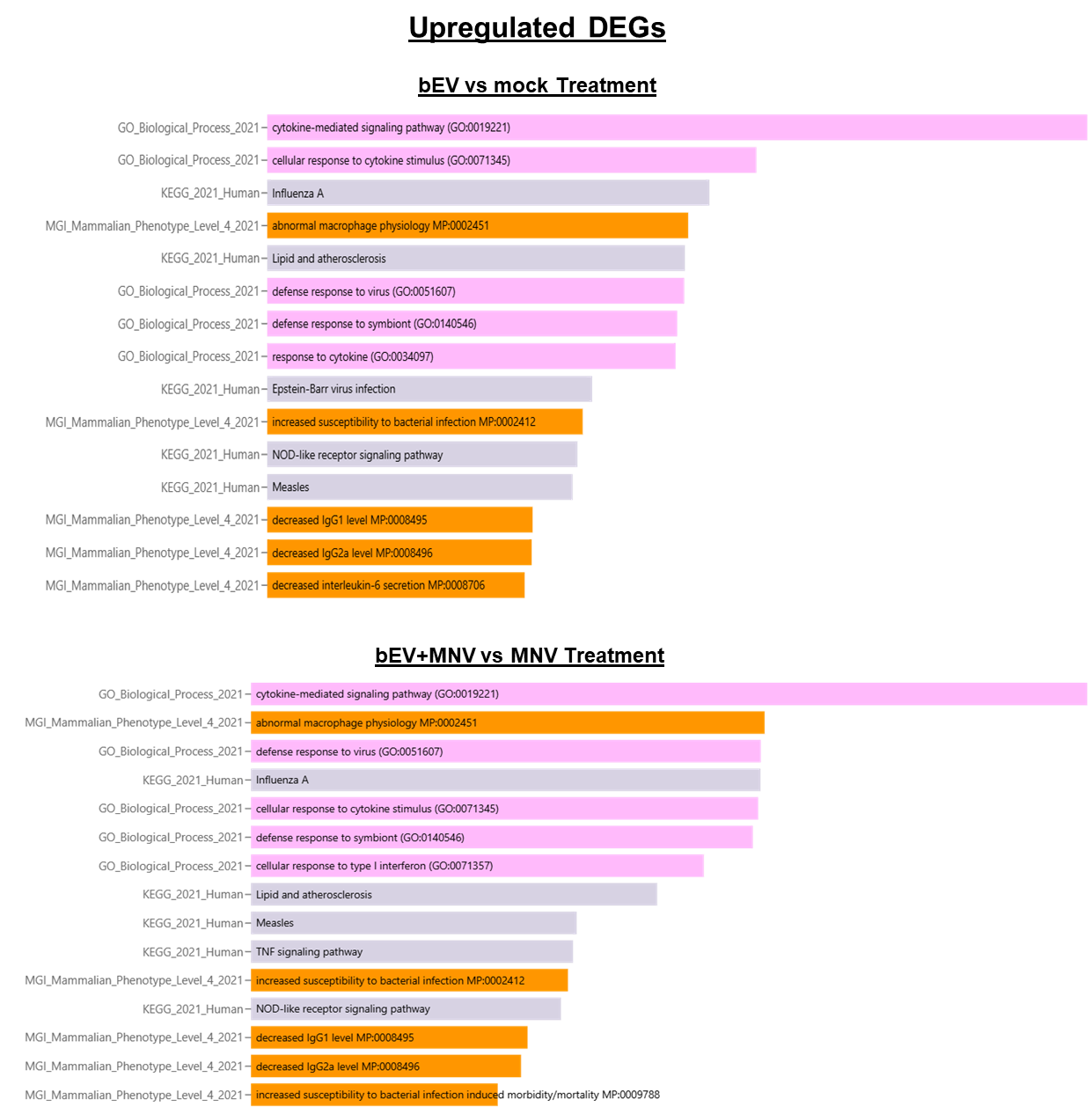


**
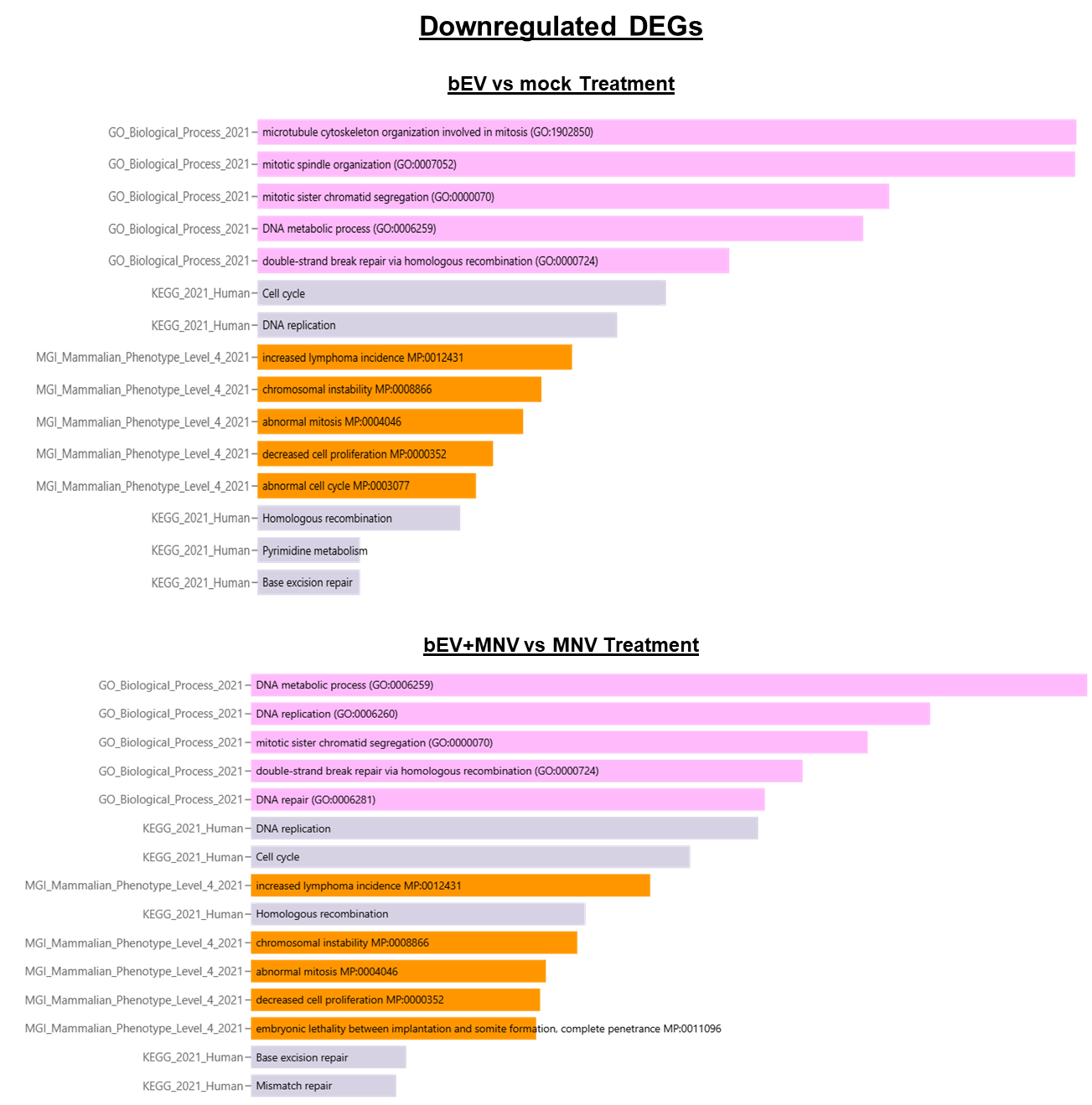
**

**Figure S4.** **Pathway analysis of scRNA-seq data expression analysis of macrophages treated with bEVs and MNV.** Lists of the top (A) upregulated and (B) downregulated DEGs were uploaded to Enrichr and pathway enrichment analysis were performed to demonstrate how bEV treatment might affect potential metabolic pathway. The top 15 up and down regulated pathways are listed for each treatment.


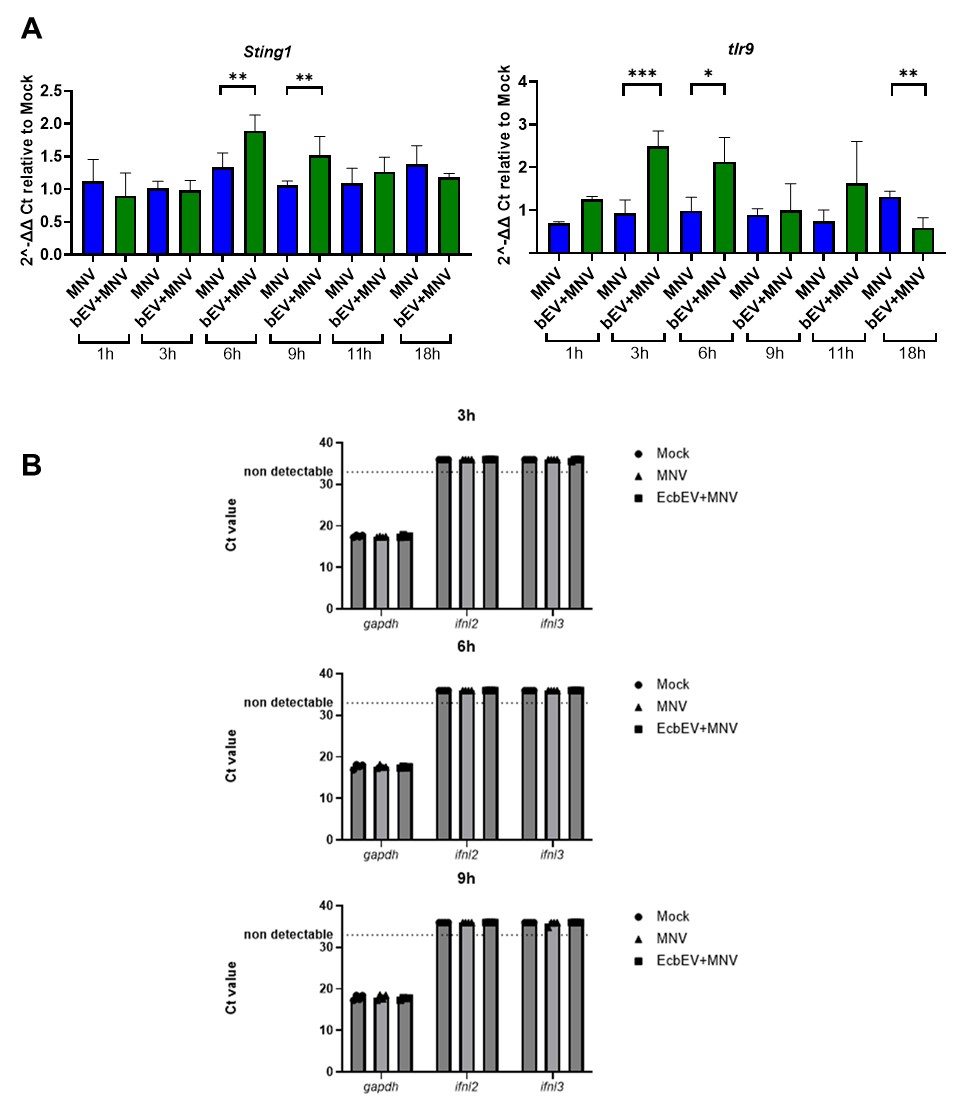


**Figure S5.** **Gene expression analysis of macrophages treated with bEVs and MNV.** (A) RAW 264.7 macrophages were infected with either MNV only or MNV+*E. cloacae* bEVs at (A) 1, 3, 6, 9, 11, 18hpi, or (B) 3, 6 and 9hpi where the Y axis denotes to raw Ct value and Ct >= 33 denotes to non-detectable value. n=4. Cells were harvested and RNA isolated for gene expression. n=6. * p< 0.05; ** p < 0.01; *** p< 0.001.

**Table S1. The top 50 variable gene features from scRNA sequencing data. Functions were obtained from Uniprot.**

| Protein | Entry ID | Function | Pubmed | UniProtKB |
| --- | --- | --- | --- | --- |
| Apba1 | B2RUJ5 | Component of the LIN-10-LIN-2-LIN-7 complex, which associates with the motor protein KIF17 to transport vesicles containing N-methyl-D-aspartate (NMDA) receptor subunit NR2B along microtubules | 10846156 |  |
| Fpr3 | O08790 | Low affinity receptor for N-formyl-methionyl peptides. Receptor for lipoxin A4. May have an olfactory function associated with the identification of pathogens or of pathogenic states. | 19387439 |  |
| Il13ra2 | O88786 | Cell surface receptor that plays a role in the regulation of IL-13-mediated responses. Functions as a decoy receptor that inhibits IL-13- and IL-4-mediated signal transduction via the JAK-STAT pathway and thereby modulates immune responses and inflammation. Serves as a functional signaling receptor for IL-13 in an alternative pathway involving AP-1 ultimately leading to the production of TGFB1. | 12642602 | Q14627 |
| Il1a | P01582 | Cytokine constitutively present intracellularly in nearly all resting non-hematopoietic cells that plays an important role in inflammation and bridges the innate and adaptive immune systems. Signaling involves the recruitment of adapter molecules such as MYD88, IRAK1 or IRAK4.Within the cell, acts as an alarmin and cell death results in its liberation in the extracellular space after disruption of the cell membrane to induce inflammation and alert the host to injury or damage. Directly senses DNA damage and acts as a signal for genotoxic stress without loss of cell integrity. | 16256210; 1386364 | P01583 |
| Saa3 | P04918 | Major acute phase reactant. Apolipoprotein of the HDL complex. In vitro exhibits antimicrobial activity against Escherichia coli, Streptococcus uberis and Pseudomonas aeruginosa. |  | Q8SQ28 |
| Plau | P06869 | Cleaves the zymogen plasminogen to form the active enzyme plasmin. |  | P00749 |
| Lif | P09056 | Can induce terminal differentiation in leukemic cells. Induction of hematopoietic differentiation in normal and myeloid leukemia cells, the induction of neuronal cell differentiation, and the stimulation of acute-phase protein synthesis in hepatocytes. |  |  |
| Mx1 | P09922 | Interferon-induced dynamin-like GTPase with antiviral activity. Inhibits FLUAV by interfering with the process of primary transcription, probably by affecting the viral polymerase function. | 17652381; 21651940 |  |
| Ccl2 | P10148 | Ligand for CCR2. Induces a strong chemotactic response and mobilization of intracellular calcium ions. Exhibits a chemotactic activity for monocytes and basophils but not neutrophils or eosinophils. | 29993042 | P13500 |
| Il1b | P10749 | Potent pro-inflammatory cytokine. Promotes Th17 differentiation of T-cells. Synergizes with IL12/interleukin-12 to induce IFNG synthesis from T-helper 1 (Th1) cells. Plays a role in angiogenesis by inducing VEGF production synergistically with TNF and IL6. Involved in transduction of inflammation downstream of pyroptosis. |  | P01584 |
| Cxcl2 | P10889 | Chemotactic for human polymorphonuclear leukocytes but does not induce chemokinesis or an oxidative burst. |  |  |
| Serpinb2 | P12388 | Inhibits urokinase-type plasminogen activator. |  |  |
| Cxcl10 | P17515 | Pro-inflammatory cytokine involved in a variety of processes such as chemotaxis, differentiation, and activation of peripheral immune cells, regulation of cell growth, apoptosis and modulation of angiostatic effects. Stimulates the activation and migration of immune cells to the infected sites . Mechanistically, binding to the CXCR3 receptor activates G protein-mediated signaling and results in downstream activation of phospholipase C-dependent pathway, an increase in intracellular calcium production and actin reorganization. In turn, recruitment of activated Th1 lymphocytes occurs at sites of inflammation. | 28623423; 18624292; 19017990; 28468883; 15456824 | P02778 |
| Slc7a2 | P18581 | Functions as a permease involved in the transport of the cationic amino acids (L-arginine, L-lysine, L-ornithine and L-homoarginine). May play a role in classical or alternative activation of macrophages via its role in arginine transport. | 8195186; 8385111; 9174363; 16670299 |  |
| Edn1 | P22387 | Endothelins are endothelium-derived vasoconstrictor peptides. Probable ligand for G-protein coupled receptors EDNRA and EDNRB which activates PTK2B, BCAR1, BCAR3 and, GTPases RAP1 and RHOA cascade in glomerular mesangial cells. Also binds the DEAR/FBXW7-AS1 receptor. Promotes mesenteric arterial wall remodeling via activation of ROCK signaling and subsequent colocalization of NFATC3 with F-actin filaments. NFATC3 then translocates to the nucleus where it subsequently promotes the transcription of the smooth muscle hypertrophy and differentiation marker ACTA2. | 16293765; 20495147; 21525433 | P05305; P09558 |
| Nos2 | P29477 | Produces nitric oxide (NO) which can mediate tumoricidal and bactericidal actions. As component of the iNOS-S100A8/9 transnitrosylase complex involved in the selective inflammatory stimulus-dependent S-nitrosylation of GAPDH implicated in regulation of the GAIT complex activity. Involved in inflammation, enhances the synthesis of pro-inflammatory mediators such as IL6 and IL8. | 7503239; 16373578 | P35228; P79290 |
| Mmp9 | P41245 | Matrix metalloproteinase that plays an essential role in local proteolysis of the extracellular matrix and in leukocyte migration (By similarity). Cleaves type IV and type V collagen into large C-terminal three quarter fragments and shorter N-terminal one quarter fragments. Degrades fibronectin but not laminin or Pz-peptide. | 8132709; 23142597; 32883094 | P14780 |
| Mxd1 | P50538 | Component of a transcriptional repressor complex together with MAX. In complex with MAX binds to the core DNA sequence 5'-CAC[GA]TG-3'. Antagonizes MYC transcriptional activity by competing with MYC for MAX binding. Binds to the TERT promoter and represses telomerase expression, possibly by interfering with MYC binding . |  | Q05195 |
| Nos2 | P60322 | Plays a key role in the sexual differentiation of germ cells by promoting the male fate but suppressing the female fate. Maintains the suppression of meiosis by preventing STRA8 expression, which is required for premeiotic DNA replication, after CYP26B1 is decreased. Regulates the localization of the CCR4-NOT deadenylation complex to P-bodies and plays a role in recruiting the complex to trigger the degradation of mRNAs involved in meiosis. Required for the maintenance of the spermatogonial stem cell population. Not essential for the assembly of P-bodies but is required for the maintenance of their normal state. | 12947200; 17138666; 18281459; 19745153; 20133598 |  |
| Fgf13 | P70377 | Microtubule-binding protein which directly binds tubulin and is involved in both polymerization and stabilization of microtubules. Regulates voltage-gated sodium channel transport and function. May also play a role in MAPK signaling. | 30679375; 11378392; 12244047; 21817159; 22726441 |  |
| Rgs16 | P97428 | Regulates G protein-coupled receptor signaling cascades . Inhibits signal transduction by increasing the GTPase activity of G protein alpha subunits, thereby driving them into their inactive GDP-bound form. Plays an important role in the phototransduction cascade by regulating the lifetime and effective concentration of activated transducin alpha. May regulate extra and intracellular mitogenic signals. | 9079700; 10373502; 8917514 |  |
| Vegfc | P97953 | Growth factor active in angiogenesis. May function in angiogenesis of the venous and lymphatic vascular systems during embryogenesis, and also in the maintenance of differentiated lymphatic endothelium in adults. Binds and activates KDR/VEGFR2 and FLT4/VEGFR3 receptors. | 9012504; 9247316 |  |
| Ccl7 | Q03366 | Chemotactic factor that attracts monocytes and eosinophils, but not neutrophils. Augments monocyte anti-tumor activity. |  |  |
| Ptgs2 | Q05769 | Dual cyclooxygenase and peroxidase in the biosynthesis pathway of prostanoids, a class of C20 oxylipins, with a particular role in the inflammatory response. Plays a role in the generation of resolution phase interaction products (resolvins) during both sterile and infectious inflammation. | 12925531; 20463020; 20810665; 21489986; 22942274 |  |
| Psd3 | Q2PFD7 | Guanine nucleotide exchange factor for ARF6. | 16707115 |  |
| Cmpk2 | Q3U5Q7 | Mitochondrial nucleotide monophosphate kinase needed for salvage dNTP synthesis that mediates immunomodulatory and antiviral activities through IFN-dependent and IFN-independent pathways. Restricts the replication of multiple viruses including flaviviruses or coronaviruses through inhibition of the viral RNA-dependent RNA polymerase activities. Is able to phosphorylate dUMP, dCMP, CMP, UMP and monophosphates of the pyrimidine nucleoside analogs ddC, dFdC, araC, BVDU and FdUrd with ATP as phosphate donor. Controls mitochondrial DNA synthesis by supplying required deoxyribonucleotides. CMPK2-dependent mitochondrial DNA synthesis is necessary for the production of oxidized mitochondrial DNA fragments after exposure to NLRP3 activators. | 30046112; 30046112; 36443312 | Q5EBM0 |
| Mx1 | Q3UD61 | Interferon-induced dynamin-like GTPase with antiviral activity |  |  |
| Myo1d | Q5SYD0 | Unconventional myosin that functions as actin-based motor protein with ATPase activity. Plays a role in endosomal protein trafficking, and especially in the transfer of cargo proteins from early to recycling endosomes. |  | F1PRN2; Q63357 |
| Cp | Q61147 | Multifunctional blue, copper-binding glycoprotein. It has ferroxidase activity without releasing radical oxygen species. It is involved in iron transport across the cell membrane. Oxidizes highly toxic ferrous ions to the ferric state for further incorporation onto apo-transferrins, catalyzes Cu(+) oxidation. Provides Cu(2+) ions for the ascorbate-mediated deaminase degradation of the heparan sulfate chains of GPC1. Has glutathione peroxidase-like activity, can remove both hydrogen peroxide and lipid hydroperoxide in the presence of thiols. Also shows NO-oxidase and NO2 synthase activities. | 29183916 | P00450; P13635 |
| Pdpn | Q62011 | Mediates effects on cell migration and adhesion through its different partners. In lymph nodes (LNs), controls fibroblastic reticular cells (FRCs) adhesion to the extracellular matrix (ECM) and contraction of the actomyosin by maintaining ERM proteins (EZR; MSN and RDX) and MYL9 activation through association with unknown transmembrane proteins. Through binding with LGALS8 may participate in connection of the lymphatic endothelium to the surrounding extracellular matrix. Controls invadopodia stability and maturation leading to efficient degradation of the extracellular matrix (ECM) in tumor cells through modulation of RHOC activity in order to activate ROCK1/ROCK2 and LIMK1/LIMK2 and inactivation of CFL1. | 12032185; 10574709; 12032185; 12654292; 14522983; 15231832; 17616532; 20110424; 25347465 | Q86YL7, |
| Ifit1 | Q64282 | Interferon-induced antiviral RNA-binding protein that specifically binds single-stranded RNA bearing a 5'-triphosphate group (PPP-RNA), thereby acting as a sensor of viral single-stranded RNAs and inhibiting expression of viral messenger RNAs. | 21085181; 22589727 |  |
| Lix1 | Q6P566 | Protein limb expression 1 |  |  |
| Nes | Q6P5H2 | Promotes the disassembly of phosphorylated vimentin intermediate filaments (IF) during mitosis and may play a role in the trafficking and distribution of IF proteins and other cellular factors to daughter cells during progenitor cell division. | 20963821 |  |
| Dcstamp | Q7TNJ0 | Probable cell surface receptor that plays several roles in cellular fusion, cell differentiation, bone and immune homeostasis. Plays a role in haematopoietic stem cell differentiation of bone marrow cells toward the myeloid lineage. Inhibits the development of neutrophilic granulocytes. Plays also a role in the regulation of dendritic cell (DC) antigen presentation activity by controlling phagocytic activity. Involved in the maintenance of immune self-tolerance and avoidance of autoimmune reactions. | 15452179; 16061724; 16937266; 17164993; 17713547; 18653699; 18952287; 20039274; 22337159 |  |
| Tafa2 | Q7TPG7 | Role as neurotrophic factor involved in neuronal survival and neurobiological functions. | 30137205 |  |
| Tnc | Q80YX1 | Ligand for integrins ITGA8:ITGB1, ITGA9:ITGB1, ITGAV:ITGB3 and ITGAV:ITGB6. | 16553788 | P24821 |
| Kirrel3 | Q8BR86 | May be involved in the hematopoietic supportive capacity of stroma cells; the secreted extracellular domain is directly responsible for supporting hematopoietic stem cells. | 23637329; 26575286 |  |
| Il33 | Q8BVZ5 | Cytokine that binds to and signals through the IL1RL1/ST2 receptor which in turn activates NF-kappa-B and MAPK signaling pathways in target cells . Involved in the maturation of Th2 cells inducing the secretion of T-helper type 2-associated cytokines. Involved in activation of mast cells, basophils, eosinophils and natural killer cells. Acts as an enhancer of polarization of alternatively activated macrophages. Acts as a chemoattractant for Th2 cells, and may function as an 'alarmin', that amplifies immune responses during tissue injury. Induces rapid UCP2-dependent mitochondrial rewiring that attenuates the generation of reactive oxygen species and preserves the integrity of Krebs cycle required for persistent production of itaconate and subsequent GATA3-dependent differentiation of inflammation-resolving alternatively activated macrophages. | 29045903; 34644537 | O95760 |
| Rsad2 | Q8CBB9 | Interferon-inducible antiviral protein which plays a major role in the cell antiviral state induced by type I and type II interferon. Catalyzes the conversion of cytidine triphosphate (CTP) to 3'-deoxy-3',4'-didehydro-CTP (ddhCTP) via a SAM-dependent radical mechanism. In turn, ddhCTP acts as a chain terminator for the RNA-dependent RNA polymerases from multiple viruses and directly inhibits viral replication. | 17686841, 19047684, 21435586, 21880757, | Q8WXG1 |
| Adgrl2 | Q8JZZ7 | Receptor probably implicated in the regulation of exocytosis. | 24273166 | O88923 |
| Cacna1d | Q99246 | Voltage-sensitive calcium channels (VSCC) mediate the entry of calcium ions into excitable cells and are also involved in a variety of calcium-dependent processes, including muscle contraction, hormone or neurotransmitter release, gene expression, cell motility, cell division and cell death. | 16354915 |  |
| Cxcl11 | Q9JHH5 | Binds to CXCR3. May play an important role in CNS diseases which involve T-cell recruitment. May play a role in skin immune responses. |  |  |
| Rbfox1 | Q9JJ43 | RNA-binding protein that regulates alternative splicing events by binding to 5'-UGCAUGU-3' elements. Prevents binding of U2AF2 to the 3'-splice site. Regulates alternative splicing of tissue-specific exons and of differentially spliced exons during erythropoiesis. | 12574126; 15824060; 16260614; 17101796 |  |
| Chst11 | Q9JME2 | Catalyzes the transfer of sulfate to position 4 of the N-acetylgalactosamine (GalNAc) residue of chondroitin. Can also sulfate Gal residues in desulfated dermatan sulfate. |  |  |
| Pde7b | Q9QXQ1 | Hydrolyzes the second messenger cAMP, which is a key regulator of many important physiological processes. | 10872825 | Q9NP56 |
| Spink5 | Q148R4 | Produces a protein called LEKT1, which controls the activity of serine peptidases |  |  |
| Lipg | Q9WVG5 | Exerts both phospholipase and triglyceride lipase activities. |  | Q9Y5X9 |
| Gm47729 | N/A | long non-coding RNA |  |  |
| Mir155hg | N/A | Regulates innate antiviral immunity by encoding long noncoding RNA-155 and microRNA-155-5p |  |  |
| GM30382 | N/A | long non-coding RNA |  |  |
| Ifit1bl1 | D3Z6F0 | Part of the IFIT family. It is predicted to bind to RNA and inhibits viral translation |  |  |
